# Co-morbidities, complications and causes of death among people with femoral neck fracture – a three-year follow-up study

**DOI:** 10.1186/s12877-016-0291-5

**Published:** 2016-06-03

**Authors:** Monica Berggren, Michael Stenvall, Undis Englund, Birgitta Olofsson, Yngve Gustafson

**Affiliations:** Department of Community Medicine and Rehabilitation, Geriatric Medicine, Umeå University, SE-901 87 Umeå, Sweden; Department of Surgical and Perioperative Sciences, Orthopaedics, Umeå University, SE-901 87 Umeå, Sweden; Department of Nursing, Umeå University, SE-901 87 Umeå, Sweden

**Keywords:** Hip fracture, Cause of death, Complications

## Abstract

**Background:**

The poor outcome after a hip fracture is not fully understood.

The aim of the study was to describe the prevalence of co-morbidities, complications and causes of death and to investigate factors that are able to predict mortality in old people with femoral neck fracture.

**Methods:**

Data was obtained from a randomized, controlled trial with a 3-year follow-up at Umeå University Hospital, Sweden, which included 199 consecutive patients with femoral neck fracture, aged ≥70 years. The participants were assessed during hospitalization and in their homes 4, 12 and 36 months after surgery. Medical records and death certificates were analysed.

**Results:**

Multivariate analysis revealed that cancer, dependence in P-ADL (Personal Activities of Daily Living), cardiovascular disease, dementia at baseline or pulmonary emboli or cardiac failure during hospitalization were all independent predictors of 3-year mortality. Seventy-nine out of 199 participants (40 %) died within 3 years. Cardiovascular events (24 %), dementia (23 %), hip-fracture (19 %) and cancer (13 %) were the most common primary causes of death. In total, 136 participants suffered at least one urinary tract infection; 114 suffered 542 falls and 37 sustained 56 new fractures, including 13 hip fractures, during follow-up.

**Conclusion:**

Old people with femoral neck fracture have multiple co-morbidities and suffer numerous complications. Thus randomized intervention studies should focus on prevention of complications that might be avoidable such as infections, heart diseases, falls and fractures.

## Background

Hip fractures are a common and major health problem among older people [1]. The annual number of such fractures in Sweden is expected to almost double during the first half of this century [2]. There is a well-established increased risk of death after hip fracture [3, 4]. It has been shown that older people have a 5- to 8-fold increased risk of dying during the first 3 months after a hip fracture [4]. Studies have been performed to optimize care of hip fracture patients and the consensus concerning preoperative management, time to surgery, operative management, surgical technique and postoperative care has led to recommendations concerning clinical care pathways and a multidisciplinary approach [5–8]. Despite research into care improvements for hip fracture patients, for example in the fields of medical and surgical care or the use of multidisciplinary teams, it has been shown that fewer than half regain their previous level of function [9] and the mortality rate has remained stable over the past 40 years [10].

A variety of postoperative complications and times to follow-up are described. One study found that 33 % of the participants had at least one complication after an operation for hip-fracture which led to prolonged hospitalization. The most common postoperative complications were delirium and infection [11]. Another study found heart failure and chest infections to be the most common postoperative complications [12], while a more recent study showed that falls, fractures and pneumonia were the most common [13].

A range of interventions to reduce the rate of in-hospital postoperative complications and mortality have been reported in the literature. One such study found that postoperative complications and 12-month mortality among community dwellers were reduced when a comprehensive multidisciplinary fast-track treatment and care program were put in place [14]. We have shown earlier that a multidisciplinary, multi-factorial rehabilitation program reduced in-hospital complications [15], including significant fewer in hospital falls but there was no difference in the number of falls during 1 year after discharge [16, 17].

Death following hip fracture has been associated with several risk factors; older age and male sex, severe systemic disease, pre-fracture functional impairment, cognitive decline, coronary heart disease and the number of co-morbidities [18–23]. The causes of excess death have been a subject of debate. One study suggests that the increased mortality is associated with postoperative complications [24], others ascribe it to pre-fracture co-morbidities together with postoperative complications [12, 19], or suggest that the co-morbidities are the underlying cause [25]. Only a few studies describe the causes of death [24, 26–29]. The most common causes of death in one autopsy study were chest infection, cardiac failure, myocardial infarction and pulmonary embolism [29]. Among nursing-home residents with hip fracture the most common causes of death were infection, dementia and cardiac events [26] and, in more recent studies, cardiac and infectious diseases [27, 28].

In spite of earlier research, the poor outcome for people with hip fracture has not improved and mortality has not been reduced. Since neither the events leading to death, nor the causes of death or the patient’s outcome after discharge have been fully investigated, we decided to explore these factors in order to discover factors that might be adjusted in order to improve outcome.

Thus, the aim of this study was to describe the prevalence of co-morbidities, complications and causes of death and to investigate factors that could predict mortality in old people with femoral neck fracture.

## Methods

### Study design

The article is based on data from a randomized controlled trial, evaluating a multidisciplinary intervention program for persons with a femoral neck fracture compared to conventional care. The intervention resulted in fewer complications during hospitalization, but there were no differences in the incidence of complications and mortality between the intervention and control groups after discharge (data not shown). The participants are analysed as one group in the present study, they were followed from admission to hospital until death, relocation or the end of 36 months of follow-up. The description of the recruitment and randomization as well as the content of the intervention has been presented in detail in earlier articles [15, 16]. All participants received oral and written information and in those cases where they were not able to answer themselves their next of kin was also asked. A written informed consent for participation in the study was required.

### Sample

**Fig. 1:**
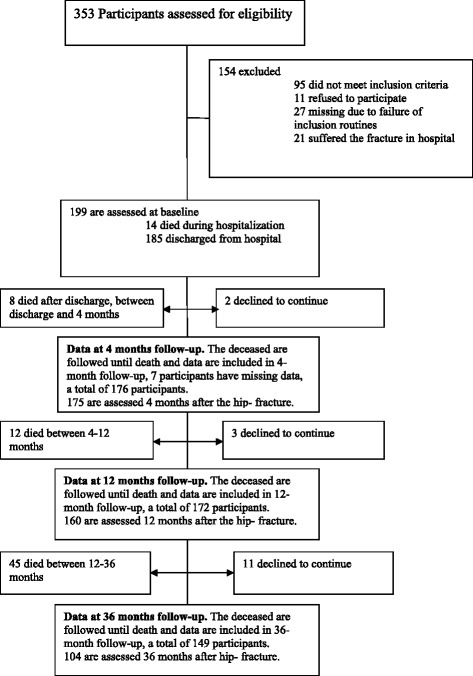
Flow chart of the results of all 353 patients with femoral-neck fractures during the study period

The study included 199 participants with femoral neck fractures (index hip fracture) aged 70 years or older, consecutively admitted to the Orthopaedic Department at the University Hospital in Umeå, Sweden, over 32 months. Flow chart of the results of all 353 patients with femoral-neck fractures during the study period is shown in Fig. 1.

Exclusion criteria were: the presence of rheumatoid arthritis, severe hip osteoarthritis, and pathological fracture, due to the planned operation methods to be used in the study. Patients with severe renal failure, those who were bedridden prior to the fracture and patients already admitted to hospital for other reasons, who then fell and suffered a hip fracture were also excluded. The surgeon decided whether or not an operation planned according to the study protocol was appropriate for the patient.

### Data collection

Trained research nurses, not involved in the patients’ medical care or nursing, assessed the patients during hospitalization and at 4, 12 and 36 months subsequently. At the follow-ups the nurse was accompanied by a physiotherapist or an occupational therapist who also made assessments. The patients’ general health was assessed before surgery, according to the American Society of Anaesthesiologists (ASA) classification [30], by the attending anaesthesiologist. ASA 1 indicates a healthy person; ASA 2, a person with a mild systemic disease; ASA 3, a person with severe systemic disease; ASA 4, a person with an incapacitating disease that is a constant threat to life; ASA 5, a moribund person who is not expected to live 24 h with or without surgery. In the analysis the ASA results were categorized as ASA 1–2 and ASA 3–4. No person classified as ASA 5 was included. All medical and social data were collected from interviews with participants, relatives and staff, and included morbidity and complications. Data were also collected from the medical and nursing records. A broad definition of complications was chosen to describe the wide spectrum of complications. The complications were classified dichotomously (present/absent) and the total number of times a complication occurred was counted. The participants with cardiac failure at baseline were judged to have cardiac failure as a complication during follow-up if they had been treated by a medical doctor due to exacerbation of the disease. Cardiac failure and myocardial infarction are referred to in the text as cardiovascular events. Infections were divided into 5 groups; chest infections, urinary tract infections, superficial and deep wound infections and other infections. A fall was defined as an incident when the participant unintentionally came to rest on the floor or ground and also included syncopal falls [31].

For those participants who died during the study, medical records were reviewed and all complications and the “history of death” were noted. If a participant died between follow-ups, complications that occurred between last follow-up and death were forwarded, which means they were registered at the next follow-up. The primary and secondary causes of death were also obtained from the death certificates. In Sweden every citizen has a unique 10-digit personal identification number which makes it possible to obtain cause and date of death from the Centre for Epidemiology (EpC), National Board of Health and Welfare, Sweden. The diagnoses are coded according to the International Classification of Diseases (ICD), which is validated and used as an international standard by the WHO (World Health Organization). In Sweden 99 % of deceased receive a diagnosis [32].

If a person died within 30 days after a fracture the primary cause of death was coded, according to international standards, as related to the trauma irrespective of the actual cause of death. For example, a myocardial infarction complicating a hip fracture would be coded as a secondary cause of death. In our analyses the causes of death were determined through assessment of the notes concerning the person’s death and the information obtained from death certificates. Fifteen of the causes of death stated in the death certificates were changed after examination of the patients’ medical records. Two of these fifteen were changed in accordance with autopsy reports, another three after more information was retrieved and a further ten were changed to secondary cause on the death certificate. For example, a participant with advanced dementia who suffered terminal pneumonia was judged to have died due to dementia rather than pneumonia.

A geriatrician, unaware of study-group allocation, analysed all assessments and documentation once the study was ended, to determine whether the participants fulfilled the DSM-IV (Diagnostic and Statistical Manual of Mental Disorders, Fourth Edition) criteria [33] for dementia, delirium and depression. The Ethics Committee of the Faculty of Medicine at Umea University approved the study (§ 00–137).

### Statistics

Pearson’s chi-square test, Fisher’s exact test and Mann–Whitney U test were used to analyse differences between the deceased and the survivors regarding complications. Univariate and multivariate Cox proportional hazard regression was used to analyse associations between baseline variables and complications during hospitalization and all-cause mortality during the 3-year follow-up. All baseline variables and complications during hospitalization associated with time to death (*p* < 0.05) in univariate analyses were included in two separate multivariate models (Model A and B in Table 1). Correlations between baseline variables and among complications during hospitalization were tested using Pearson’s and Spearman’s coefficients; the covariate Living in residential care facilities before fracture was removed due to its strong correlation with Dependence in personal activities of daily living (P-ADL) (*r* > 0.6). Stepwise backward deletion was performed manually until only significant variables remained in the models.

**Table 1:** Baseline characteristics and complications among participants and differences among deceased and survivors. Univariate and multivariate cox regression analyses

	All cases	Deceased within 3 years	Survivors	Univariate	Multivariate		
	*n* = 199 (%)	*n* = 79	*n* = 120	P^a^	P^b^	HR	CI
Baseline characteristics:					Model A.		
Age, mean ± SD	82.2 ± 6.2	82.9 ± 5.8	81.7 ± 6.5	0.233	0.435	1.016	0.977–1.056
Female	148 (74 %)	53	95	0.029	0.052	0.604	0.363–1.005
Living in residential care facilities	73 (37 %)	37	36	0.028			
Living alone	146 (74 %)	59	87	0.696			
Current smoker (*n* = 169)	6 (4 %)	2	4	0.955			
Dependence in walking (*n* = 195)	25 (13 %)	15	10	0.004			
Dependence in P-ADL	115 (58 %)	59	56	<0.001	0.005	2.379	1.300–4.354
Cancer (*n* = 194)	29 (15 %)	21	8	<0.001	<0.001	3.449	2.020–5.890
Cardiovascular disease (*n* = 194)	110 (57 %)	55	55	<0.001	0.004	2.177	1.287–3.682
Dementia	64 (32 %)	37	27	0.001	0.016	1.921	1.131–3.262
Depression (*n* = 197)	78 (40 %)	39	39	0.040			
Diabetes (*n* = 197)	40 (20 %)	22	18	0.059			
Kidney disease (*n* = 194)	21 (11 %)	10	11	0.321			
Pulmonary disease (*n* = 194)	33 (17 %)	17	16	0.049			
Stroke (*n* = 195)	49 (25 %)	23	26	0.184			
ASA grade 3–4 (*n* = 197)	109 (55 %)	51	58	0.007			
Internal fixation	69 (35 %)	33	36	0.420			
Hemiarthroplasty	111 (56 %)	37	74	0.228			
Dynamic hip screw	17 (9 %)	7	10	0.998			
Other	1 (0.5 %)	1	0	0.345			
No of. comorbidities:							
0	31 (16 %)	3	28	<0.001			
1	37 (19 %)	7	30	0.260			
2	52 (26 %)	25	27	0.005			
≥3	79 (40 %)	44 (56 %)	35 (29 %)	0.001			
Complications during hospitalization:					Model B.		
Pneumonia/chest infection	8	6	2	0.001	0.021	2.916	1.175–7.237
Urinary tract infection	82	38	44	0.241			
Other infection	34	15	19	0.528			
Wound infection	0	0	0				
Deep wound infection	1	1	0	0.054			
Cardiac failure	17	12	5	0.001	0.010	2.457	1.242–4.858
Myocardial infarction	6	6	0	0.052			
Deep vein thrombosis	1	1	0	0.050			
Pulmonary embolism	1	1	0	0.002	0.001	33.219	3.961–278.570
Stroke	3	2	1	0.072			
Cancer	3	2	1	0.120			
Gastric ulcer	7	3	4	0.538			
Delirium	129	63	70	0.005	0.018	2.006	1.127–3.570
Fallers	38	19	19	0.267			
Number of falls	78	41	37	0.262			
Fracture	4	2	2	0.609			
Luxation	6	3	3	0.416			
Reoperation	8	3	5	0.926			
Decubital ulcers	30	16	14	0.044			
Combining baseline and complication factors:					Model C.		
Age					0.705	1.008	0.968–1.049
Female					0.118	0.655	0.386–1.113
Dependence in P-ADL					0.007	2.362	1.271–4.387
Cancer					<0.001	3.393	1.959–5.877
Cardiovascular disease					0.013	2.026	1.160–3.539
Dementia					0.023	1.883	1.091–3.250
Pneumonia/chest infection							
Cardiac failure					0.018	2.221	1.148–4.294
Pulmonary embolism					<0.001	69.396	7.107–677.632
Delirium							

*SD* Standard deviation

*P-ADL* Personal Activities of Daily Living

*ASA* American Society of Anaesthesiologists classification

*HR* Hazard ratio

*CI* 95 % Confidence interval

^a^ p according to univariate cox regression model

^b^ p according to multivariate cox regression model

The proportionality of hazards was tested using Schoenfeld residuals and time-dependent variables in extended Cox regression models [34]. A final multivariate model adjusted for age, sex and remaining significant variables from the two separate multivariate analyses was performed (Model C in Table 1). All calculations were carried out using SPSS v 23. All statistical tests were 2-tailed and a *p*-value <0.05 was considered statistically significant.

## Results

### Predictors of mortality

A history of cardiovascular disease, cancer, dependence in P-ADL, dementia, having three or more co-morbidities, dependence in walking, having an ASA score of 3 or higher, living in residential care facilities, male gender, depression and pulmonary disease were all independently associated with time to death in univariate Cox regression analyses (Table 1). The results from multivariate Cox regression analyses are also displayed in the columns to the right of Table 1. The hazard ratios (HR) and 95 % confidence intervals (CI) are presented for the significant variables remaining in the multivariate models. When analysing associations between baseline variables and time to death we found that cancer, cardiovascular disease, dependence in P-ADL and dementia were associated with time to death in a Cox proportional hazard regression model adjusted for age and sex (Model A). In a second model (Model B.) complications during hospitalization were analysed; pulmonary emboli, pneumonia, cardiac failure and delirium were all associated with time to death. In the final model (Model C.) all significant variables in the two multivariate models above were combined; pulmonary emboli (HR 69.396, CI: 7.107–677.632), cancer (HR 3.393, CI: 1.959–5.877), cardiac failure (HR 2.221, CI: 1.148–4.294), cardiovascular disease (HR 2.026, CI: 1.160–3.539), dementia (HR 1.883, CI: 1.091–3.250) and dependence in P-ADL (HR 2.362, CI: 1.271–4.387) remained independently associated with all-cause mortality adjusted for age and gender.

### Co-morbidities and baseline characteristics

The majority of the participants were women, living alone and independently; they walked independently indoors but only four out of ten were independent in P-ADL before the index hip fracture. Fifty-seven percent had a history of cardiovascular disease and 55 % had an ASA score of 3 or higher at baseline. The deceased had three or more co-morbidities in 56 % of the cases at baseline compared to 29 % among the survivors (Table 1).

### Deaths

Seventy-nine out of 199 participants (40 %) died. Mortality was 13/199 (6 %) at 30 days and 34/199 (17 %) at 1 year after the index hip fracture. Twenty-six out of 51 (51 %) men died compared to 53/148 (36 %) women. After discharge, a total of 65 participants died during the 3 years of follow-up (Table 2).

**Table 2:** Primary causes of death

Cause of death	During hospitalization	Between discharge and 4 months	Between 4 and 12 months	Between 12 and 36 months	Total
Cardiovascular	0	1	2	16	19
Dementia	0	0	4	14	18
Hip fracture	11	3	0	1	15
Cancer	0	2	3	5	10
Cerebrovascular	0	0	1	3	4
Infection	1	0	0	1	2
Deep infection	0	0	0	2	2
Renal failure	1	0	1	0	2
Fracture	0	1	1	0	2
Gastrointestinal bleeding	1	0	0	0	1
Ruptured aortic aneurysm	0	0	0	1	1
Parkinson’s disease	0	0	0	1	1
Pancreatitis	0	0	0	1	1
Gangrene	0	1	0	0	1
Sum	14	8	12	45	79
Autopsy	7	2	1	4	15

### Causes of death

In this study 13 participants died within 30 days of sustaining the index hip fracture. Ten of these 13 died during hospitalization: one before the operation, due to myocardial infarction; two on the day of operation, due to cardiovascular events; two the day after the operation (one due to gastrointestinal bleeding and one to myocardial infarction); one suffered a cerebrovascular event during the operation and never regained consciousness; one suffered a myocardial infarction after a week; two died of pneumonia and one died due to an intestinal infarction. The participants who died during hospitalization due to a cardiovascular event all had a history of cardiovascular disease. The three participants who died after discharge but within 30 days of the index hip fracture did so due to a cerebrovascular event, a myocardial infarction and a pulmonary emboli, respectively. The secondary cause of death within 30 days of the index fracture was due to a cardiovascular event in 6/13 (46 %) cases.

In addition four participants died within 30 days after sustaining a new fracture. One of these participants fell and suffered a new hip fracture while still in hospital and then died due to an infection. After discharge one participant suffered a hip fracture and died due to pneumonia, one sustained a head trauma with a face fracture and died due to intracerebral bleeding and one had a knee fracture and died due to rupture of the aorta. Cardiovascular events (19/65), Dementia (18/65), cancer (10/65), fractures (6/65) and cerebrovascular events (4/65) were the most common primary causes of death after discharge (Table 2).

### Complications

In total 166 participants suffered from 583 infections, including 136 participants who suffered 363 urinary tract infections. Seventy-four participants suffered 111 cardiovascular events during the 3 years following the hip fracture. One-hundred and fourteen participants suffered 542 falls. Thirty-seven participants suffered 56 new fractures, including 13 new hip fractures, seven wrist fractures and seven rib fractures. Forty-nine participants suffered 60 decubital ulcers. During follow-up 96 participants had 234 hospital admissions with a total of 3984 days spent in hospital. Infections and cardiovascular events were more common after discharge among those who died (Table 3).

**Table 3:** Complications after femoral neck fracture occurring between discharge and until three year follow-up, differences between deceased and survivors

Complications	Between discharge and 4 months (*n* = 176)	Between 4 and 12 months (*n* = 172)	Between 12 and 36 months (*n* = 149)
Pneumonia/chest infection	5	8*	21*
Urinary tract infection	51*	59	78
Other infection	34	41	56
Wound infection	3	0	0
Deep wound infection	2	0	2
Cardiac failure	18*	17*	30*
Myocardial infarction	5	6*	12*
Deep vein thrombosis	1	0	1
Pulmonary embolism	1	1	2
Stroke	2	8	10
Cancer	0	4	4
Gastric ulcer	2	2	7
Fallers	53	61	66
Number of falls	126	130	208
Number of fractures	6	17	29
Luxation	3	2	4
Reoperation	3	2	7
Decubital ulcers	9	10	11
Number of participants hospitalized	25*	48*	64
Number of hospitalizations	29*	66*	139*
Number of days in hospital	413*	1134*	2437*
Median days in hospital, IQR	9.0 (5.0–18.0)	15.0 (4.0–31.2)	23.5 (9.2–48.8)

* = *p* < 0,05 according to Pearson’s chi-square, Fisher’s exact test or Mann–Whitney U test as appropriate, when comparing deceased and survivors

*IQR* Inter Quartile Rate

## Discussion

This study shows that both co-morbidities at baseline and complications during hospitalization are associated with mortality. Cancer, dependence in P-ADL, cardiovascular disease and dementia at baseline, and pulmonary emboli and cardiac failure during hospitalization were all independent predictors of mortality. Forty percent had died after 3 years despite the exclusion of those who were bedridden, had severe renal failure or pathological fractures. The most common primary causes of death were cardiovascular events, dementia, fractures, cancer and cerebrovascular events. The participants had several co-morbidities and suffered numerous complications such as infections, falls and fractures, cardiovascular events, delirium, and pressure ulcers.

When analysing factors associated with death we found that both comorbidities and post-operative complications were of significance, a finding which is also verified in a recent review using data from a National Trauma Data Bank [35]. A study by Roche et al. [12] found that age, male sex, cancer, chest infection, cardiac failure and stroke could predict mortality but they did not include dementia or functional measurements in their model. Dementia, however, is common among hip fracture patients and was thus included in the present study. Dementia is also found to be a risk factor for death and in another large cohort study by Petersen et al. [19] age, cardiac complications and dementia were associated with mortality at 12 months but malignancies, cardiovascular disease and measurements of function were not included. In a large cohort study by Castronuovo et al. [36] heart disease was a risk factor for 30-days mortality but not during follow-up though no complications were included in the study.

A reduced mental and medical status, and a poor physical ability at baseline were found among the deceased in the current study, similar to the results of a Norwegian study [23], although those living in nursing homes and patients who did not pass a mental status test had been excluded. Such patients were not excluded in the present study. Among those who died, 56 % had three or more co-morbidities at baseline and 65 % had an ASA score of 3 or higher. The number of co-morbidities and poor pre-fracture status might be indicators of frailty. It has been shown that the (ASA) classification of medical co-morbidities is strongly associated with medical problems in the perioperative period [37] and an earlier Swedish study shows that a high ASA score is a factor associated with mortality [21]. A cardiovascular disease is a strong predictor of post-operative cardiac failure, according to Roche et al. [12]. All participants in the present study who died during hospitalization due to a cardiovascular events had a pre-fracture cardiovascular disease.

Early death within 30 days after admission to hospital due to a hip fracture has recently been described in a study that examines post-mortem reports, where respiratory infections and cardiovascular disease were the main causes of death [28]. These results are in line with earlier studies [24, 29] and our study shows a similar result as 46 % of early deaths were due to a cardiovascular event. During follow-up cardiovascular events, dementia and cancer were the most common causes of death in the present study, which is partly consistent with earlier studies [26, 27, 38]. The difference in the prevalence of infection as a cause might be due to the manner in which assessment of the causes of death was determined in the present study, as described above in the method section. There might also be an under diagnosis of dementia among many old people [39]. The participants in the present study were cognitively tested during hospitalization and at 4, 12 and 36 months.

Our study confirms that complications among people suffering from a hip fracture are numerous, both early post-operatively and during follow-up. In-hospital complications among hip fracture patients, such as urinary tract infections, delirium, decubital ulcers and falls, can be successfully prevented and treated [14, 15] but as far as we know few intervention studies have succeeded in preventing cardiac complications [40–42].

Since we can now identify the most vulnerable patients, the focus in further research should be on prevention of infections and heart diseases in early postoperative care since they might be avoidable. These findings are in line with Petersen et al. [19] who concluded that cardiac complications constituted an important risk factor that might be modified and with Roche et al. [12] who emphasized the need for medical assessment among those with heart failure and chest infection.

In addition, prevention should also focus on the numerous complications that occur after discharge from hospital. General complications were associated with loss of function in a recent study by Hansson et al. [13] Improved rehabilitation after stroke, including treatment of underlying comorbidities as well as secondary prevention, has increased the survival rate after stroke over the last few decades [43] while mortality after femoral neck fracture has remained constant. Treatment of risk factors for stroke and myocardial infarction as well as secondary prevention are currently well established in routine care. The 56 new fractures that occurred in the present sample indicate that fracture prevention also needs to become a part of routine care aimed at reducing fracture rates and mortality.

Since hip fracture is, in many cases, an event that signals a systemic decline in the person’s health, it is crucial to preserve and improve the clinical care pathways to ensure optimal recovery and survival for these patients. The present study will, hopefully, contribute to the knowledge available concerning the causes of death and highlight the potentially modifiable/preventable complications that we need to focus on in the future.

The strength of our study is that we have systematically analysed all complications and the secondary causes of death among people with hip fracture, which no other study has reported to our knowledge. Differences in the present results compared to other studies might be due to the definitions applied to complications and co-morbidities and to the choice of exclusion criteria. In the future a more standardized description of the samples would facilitate comparison. In the present study we tried to obtain information about all events that the participants experienced and that led to death. An underlying cause of death is defined as “the disease or injury which initiated the train of morbid events leading directly to death, or the circumstances of the accident or violence which produced the fatal injury”, in accordance with the rules of the ICD. Although the intention of the ICD is to provide a standard means of recording underlying causes of death, comparison of cause-of-death data over time and across countries should be undertaken with caution. The rules for selecting the underlying cause of death have been re-evaluated and sometimes changed. Incorrect or incomplete death certificates, misinterpretation of ICD rules for selection of the underlying cause, and variations in the use of coding categories for unknown and ill-defined causes might all occur, according to the WHO.

There are some limitations to the present study. The sample was relatively small and the participants were only assessed three times over 3 years after discharge and there are certainly complications that were missed, despite the thorough reviews of the participants’ medical records. As people with vertebral fractures and rib fractures do not always seek medical care such fractures are poorly documented. The number of vertebral and rib fractures has probably been underestimated in our study since x-rays were not routinely taken during follow-up. Suffering pulmonary emboli is a serious condition and the HR for pulmonary emboli in the multivariate analyse should be interpreted with precaution as only one person in this sample had an emboli and died soon after the fracture.

## Conclusion

Old people with femoral neck fracture have multiple co-morbidities and suffer numerous complications which might lead to death, both during hospitalization and after discharge. Thus randomized intervention studies should focus on prevention of complications that might be avoidable such as infections, heart diseases, falls and fractures.

## Abbreviations

ASA, American Society of Anaesthesiologists classification; CI, 95 % confidence intervals; DSM-IV, diagnostic and statistical manual of mental disorders, fourth edition; EpC, centre for epidemiology; HR, hazard ratios; ICD, International Classification of Diseases; P-ADL, personal activities of daily living; WHO, World Health Organization.
